# A standardized extract of *Centella asiatica* (ECa 233) prevents temporomandibular joint osteoarthritis by modulating the expression of local inflammatory mediators in mice

**DOI:** 10.1590/1678-7757-2021-0329

**Published:** 2021-10-14

**Authors:** Nattapon ROTPENPIAN, Tawepong ARAYAPISIT, Atitaya ROUMWONG, Narawut PAKAPROT, Mayuree TANTISIRA, Aree WANASUNTRONWONG

**Affiliations:** 1 Faculty of Dentistry Prince of Songkla University Songkhla Thailand Faculty of Dentistry, Prince of Songkla University, Songkhla, Thailand.; 2 Faculty of Dentistry Mahidol University Bangkok Thailand Faculty of Dentistry, Mahidol University, Bangkok, Thailand.; 3 Faculty of Medicine Chulalongkorn University Bangkok Thailand Faculty of Medicine, Chulalongkorn University, Bangkok, Thailand.; 4 Faculty of Medicine Siriraj Hospital Mahidol University Bangkok Thailand Faculty of Medicine, Siriraj Hospital, Mahidol University, Bangkok, Thailand.; 5 Faculty of Pharmaceutical Sciences Burapha University Chonburi Thailand Faculty of Pharmaceutical Sciences, Burapha University, Chonburi, Thailand.

**Keywords:** Temporomandibular joint osteoarthritis, Complete freund adjuvant, ASIC3, CGRP, TRPV1, ECa 233

## Abstract

**Objectives:**

To investigate the effect of a standardized extract of *Centella asiatica* (ECa 233), which has anti-inflammatory properties, on the local expression of the transient receptor potential vanilloid 1 (TRPV1), the acid-sensing ion channel subunit 3 (ASIC3), and the calcitonin gene-related peptide (CGRP) in the temporomandibular joint (TMJ) structure 21 days after injecting the TMJ with complete Freund’s adjuvant (CFA).

**Methodology:**

A mouse model was induced by analyzing the CFA-injected TMJ on days 7, 14, and 21. We assessed TMJ histology by the osteoarthritis cartilage grade score. Then, we observed the effect of different ECa 233 concentrations (30, 100, and 300 mg/kg) and of 140 mg/kg ibuprofen doses on TRPV1, ASIC3, and CGRP local expression on day 21.

**Results:**

Osteoarthritis cartilage scores were 1.17±0.37 and 3.83±0.68 on days 14 and 21, respectively, in the CFA group (n=5). On day 21, TRPV1, ASIC3, and CGRP expression significantly increased in the CFA group. In the ibuprofen-treated group, TRPV1 expression significantly decreased, but ASIC3 and CGRP showed no significant difference. All ECa 233 doses reduced TRPV1 expression, but the 100 mg/kg ECa 233 dose significantly decreased ASIC3 expression.

**Conclusions:**

TRPV1, ASIC3, and CGRP expression increased in mice with TMJ-OA on day 21. All ECa 233 and ibuprofen doses inhibited pathogenesis by modulating the local expression of TRPV1 and ASIC3. Therefore, ECa 233 was more effective than ibuprofen.

## Introduction

Temporomandibular joint osteoarthritis (TMJ-OA) is a degenerative joint disease characterized by varying degrees of inflammation, destruction of the articular cartilage, and resorption of the subchondral bone^1^. It occurs at every age, but especially in people above 40 years of age, and is usually detected in patients who require dental treatment for TMJ-OA pain.^2,3^ The structure of the TMJ is composed of synovial tissue, bones, nerves, and blood vessels. Thus, TMJ injuries damage several tissues.

TRPV1 is a polymodal, nonselective cation channel and a member of the transient receptor potential vanilloid family.^4^ It is commonly expressed in sensory neurons innervating joints, especially in articular tissues.^5^ A previous study reported that the total protein levels of TRPV1 in osteoarthritis (OA) were higher than those found in healthy joints.^6^

ASIC3 is a sodium-selective ion channel activated by low extracellular pH and a member of the degenerin sodium channel superfamily. It is a pain modulator expressed in the knee joint, strongly correlated with weight-bearing pain and secondary hyperalgesia.^7^ In a hind-paw osteoarthritis model, ASIC3 was expressed in dorsal root ganglia after 21 days.^8^ Furthermore, ASIC3 can effectively transduce the prolonged tissue acidosis occurring in osteoarthritis pathology.^9^

Synthesis of the calcitonin gene-related peptide (CGRP) is activated by nerve damage and enhanced by inflammatory mediators, such as bradykinin, serotonin, and interleukins (IL) (IL-6, IL-1β, IL-17, and IL-22)— released after tissue injury—especially in the cell membrane of chondrocytes.^7-9^Moreover, CGRP also promotes TRPV1 upregulation^9,10^ and ASIC3 expression.^7,10^

TRPV1 and ASIC3 are inflammatory markers triggered by compression injuries, which are the main causes of TMJ-OA.^3,4^ CGRP also directly enhances both TRPV1 and ASIC3. In this study, we investigated whether the local expression of these three proteins in TMJ-OA could be used as a marker for target treatment.

Although nonsteroidal anti-inflammatory drugs (NSAIDs) effectively relieve pain and minimize inflammation in TMJ-OA, they may induce peptic ulcers in the gastric epithelium and increase the risk of cardiovascular diseases when used for long periods.^11^ Therefore, alternative drugs are necessary and should be investigated.

Our previous study showed that inflammation, which led to TMJ-OA, 21 days after we injected complete Freund’s adjuvant (CFA) in the TMJ of mice. Microcomputed tomography showed significant anatomical changes to the condylar head of the joint, indicating bone deformation.^12^ Therefore, condylar bone deterioration starts on day 21 and can then be diagnosed as TMJ-OA.

*Centella asiatica* is a herb that has been used as a medicine for decades in many countries, such as India, China, and even Thailand. *C. asiatica* has many interesting pharmacological properties, such as rapid wound-healing, antioxidant, and anti-inflammatory properties. Previous studies analyzed *C. asiatica* as a standardized pure extract called ECa 233 and found it to be minimally toxic. Its active ingredient is triterpenoid glycoside (85%), divided into madecassoside (53%) and asiaticoside (32%); in a ratio of 1.5:0.5.^13^ ECa 233 can suppress many inflammatory mediators, including reactive oxygen species, nitric oxide, inducible nitric oxide synthase, cyclooxygenase (COX)-2, tumor necrosis factor (TNF)-α, and IL-1β.^13,14^ Bobade, et al.^15^ (2015) reported the therapeutic effect of asiaticoside, which can decrease bradykinin levels by 5HT1A/B agonist action.^15^ Bradykinin is one of the mediators that can elevate CGRP , TRPV1, and ASIC3 expression levels.^15,16^ Therefore, the asiaticoside in ECa 233 indirectly inhibits CGRP, TRPV1, and ASIC3 expression via bradykinin reduction. However, ECa 233 protective effects against the local inflammatory mediators CGRP, ASIC3, and TRPV1—strongly associated with CFA-induced TMJ-OA —remain unreported.

Therefore, this study aims to investigate the effect of ECa 233 on the local expression of TRPV1, ASIC3, and CGRP in the TMJ 21 days after CFA-induced TMJ-OA.

## Methodology

### Study groups and experimental design

This study was approved by the Animal Care and Use Committee of the Faculty of Medicine Siriraj Hospital at the Mahidol University (SI-ACUP014/2561), complying with the Animal Research: Reporting of *In Vivo* Experiments guidelines for the use of animals in research. Sample size aimed at 80% statistical power (1-β) with a 95% confidence interval (α=0.05).^12^ The mice were divided into six groups: sham, CFA, ibuprofen (140 mg/kg), and groups treated with different ECa 233 concentrations (30, 100, and 300 mg/kg). Five animals were required for each group. Adult male mice (six week-olds weighing between 28 and 32 grams each) from the Institute of Cancer Research mice (ICR mice) were used, like previous studies on the CFA-induced TMJ-OA model. Five mice were housed in controlled temperature and humidity (22°C±2°C, and 45%±15%, respectively) cages under a 12-hour light/dark cycle.

### TMJ-OA model induction

Each mouse was intraperitoneally anesthetized with sodium pentobarbital (60 mg/kg). To induce TMJ-OA, 10 µl of 1 mg/ml CFA (F5881; Sigma-Aldrich, St. Louis, MO, USA) were dissolved in a normal saline solution (1:1) and the final solution was injected into the right TMJ of mice. The anatomical landmark of the injection was prescribed in previous studies.^12,17^ The hair around the TMJ were trimmed in order to identify them by palpation. Then, a 30-gauge needle was inserted through the facial skin, the edge of the arch, and into the TMJ space.^12^

### Administration of a standardized *C. asiatica* extract (ECa 233)

ECa 233 was kindly supplied by Siam Herbal Innovation Co. Ltd. (Lot No. ARA051401). The sham and CFA groups were given 0.3 ml of 0.5% carboxymethyl cellulose. The ECa 233-treated group received a 0.3 ml dose of ECa 233 (dissolved in 0.5% carboxymethyl cellulose) at 30, 100, or 300 mg/kg concentrations via oral gavage every day after the CFA induction for 21 consecutive days.

### The structure of the TMJ

On days 7, 14, and 21, the sham and CFA groups were deeply anesthetized and transcardially perfused with 250 ml of ice-cold phosphate-buffered saline (pH 7.4). Then, they were decapitated. The skulls were immersed for 7 days in 4% paraformaldehyde in 0.1 M phosphate-buffered saline solution (pH 7.4). Then, the skin of the skulls was removed and the cervical bones were open. Thereafter, the right TMJ was dissected and decalcified in 10% formic acid for 7 days before being embedded in paraffin. Using a microtome, the TMJ structures were sagittally sliced; including the condylar head, articular disk, and temporal bone into 10 µm sections (three sections for each mice) and stained them with hematoxylin and eosin.^18^

The TMJ sections were collected at intervals of approximately one every 10, so that the surface changes to the TMJ by hematoxylin/eosin staining could be observed. Furthermore, the degree of joint degeneration was assessed and graded individually via the following criteria of the osteoarthritis cartilage histopathology assessment system:^20,21^ intact surface/intact cartilage morphology =0, intact surface with superficial abrasion =1, surface discontinuity =2, vertical fissures or clefts =3, surface erosion =4, sclerotic bone within a denuded surface =5, and deformation =6.^12,19^

### Immunohistochemistry staining of TRPV1, ASIC3, and CGRP expression on day 21 in the condylar head of the TMJ

Every 10^th^ section of TMJ were processed to immunohistochemically examine ASIC3, CGRP, and TRPV1 expression. Since positive and negative controls were used in our staining to validate the specificity of the primary antibody; sections were immunolabeled with rabbit polyclonal TRPV1, ASIC3, and CGRP diluted to 1:200 (SC-52; Santa Cruz Biotech, Dallas, TX, USA), subjected to Dako EnVision and Peroxidase (DC EnVision System, HRP, CA, USA), and examined by light microscopy (Olympus, Tokyo, Japan). After immunohistochemistry staining, every sample was randomly distributed to the investigators. Thus, we received blind samples (single-blind technique) to eliminate data analysis biases. Our data were analyzed by the ImageJ software.

The appropriate location to analyze the quantities of molecules were selected, in order to detect proteins in the TMJ tissues. The condylar cartilage was analyze, since chondrocytes and nerve endings have exposed ASIC3 and TRPV1 on their surfaces. CGRP was also secreted from nerve endings, inducing vasodilation and immune cell extravasation.

Having detected TRPV1, ASIC3, and CGRP on our immunohistochemistry staining, the exact location of the condylar cartilage should be determined by comparing the immunohistochemistry-stained pictures to gross anatomy pictures. The parameter *area* indicated the distribution of ASIC3, CGRP, and TRPV1 in the condylar cartilage. The percentage of the stained area was determined by totaling the proportion of the protein-positive staining area and the condylar cartilage area, and multiplying them by 100.

After finding the protein-positive stained area, it was necessary to evaluate the color value of the picture background. Therefore, the values were measured to randomly select three non-stained areas on the “articular disk,” those values were recorded, and used to calculate the mean value. Then, the area whose color value was higher than the background value was selected. Thus, reliable percent values of the protein-positive stained area were obtained.

### Data analysis

All statistical data were analyzed by the Statistical Package for the Social Sciences version 26.0 (IBM, Chicago, IL, USA). Data are expressed as the mean ± standard error. Normal data distribution was examined by the Kolmogorov-Smirnov test. The sham and CFA groups were compared via the independent *t*-test. We compared the differences between groups by the one-way analysis of variance (ANOVA) and the Dunnett’s test (*post hoc* test). A *p* value of less than 0.05 indicated statistical significance.

## Results

### ECa 233 prevented the development of TMJ-OA

Table 1 summarizes the graded score of the right-side TMJ-OA according to the osteoarthritis cartilage histopathology assessment system. The condylar surface of the CFA group gradually deteriorated. On day 21, the graded score of the CFA group was 3.83±0.69 (n=5). Table 2 lists the graded scores of the osteoarthritis cartilage of all ECa 233-treated groups on day 21. No changes were observed in the sham group or the ECa 233-treated groups.

**Table 1:** Graded scores of the temporomandibular joint osteoarthritis in the sham and complete Freund’s adjuvant (CFA) groups on different days according to the osteoarthritis cartilage histopathology assessment system (n=5 per group)

Grade	Sham	CFA (Mean ± SD)
Day 7	0	0
Day 14	0	1.17±0.37
Day 21	0	3.83±0.69

**Table 2:** Graded scores of the temporomandibular joint osteoarthritis in the different groups after ECa 233 administration

Group (n = 5 per group)	Graded score (Mean ± SD)
Sham	0
CFA	3.84±0.68
Ibuprofen	0.67±0.41
ECa 233, 30 mg/kg	0.84±0.68
ECa 233, 100 mg/kg	0.67±0.42
ECa 233, 300 mg/kg	1.00±0.82

### ECa 233 reduced ASIC3, CGRP, and TRPV1 expression in the TMJ 21 days after CFA injection

**Figure 1:**
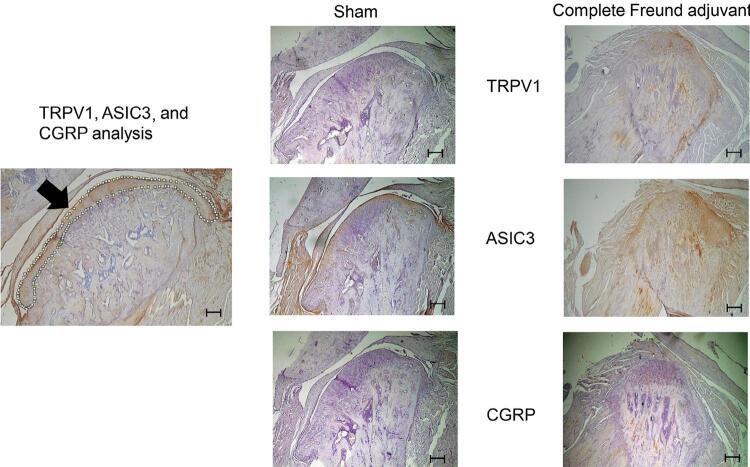
Transient receptor potential vanilloid 1 (TRPV1), acid-sensing ion channel subunit 3 (ASIC3), and calcitonin gene-related peptide (CGRP) in the sham and complete Freund’s adjuvant (CFA) groups, with a 200 µm scale bar. The small white circles pointed by the arrow on the left figure indicate the condylar head of the temporomandibular joint (TMJ), used in the analysis of all experiments

**Figure 2:**
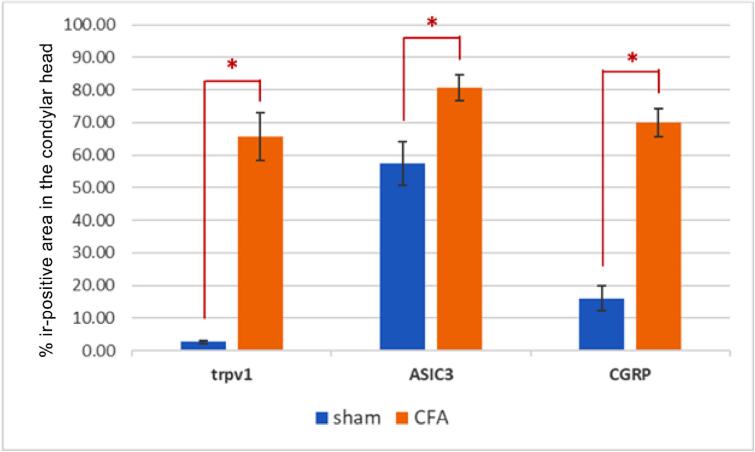
Graph of the percentage of the ir-positive area in the condylar head of the temporomandibular joint in the sham and complete Freund’s adjuvant (CFA) groups according to protein expression. Independent t-test, n=5, p≤ 0.05

On day 21, the CFA group showed increased expression of TRPV1, ASIC3 and CGRP, as observed on the condylar head (independent *t*-test, n=5, *p*<0.05). Thus, the expression of these proteins was associated with TMJ-OA evolution (Figure 1 and 2).

**Figure 3:**
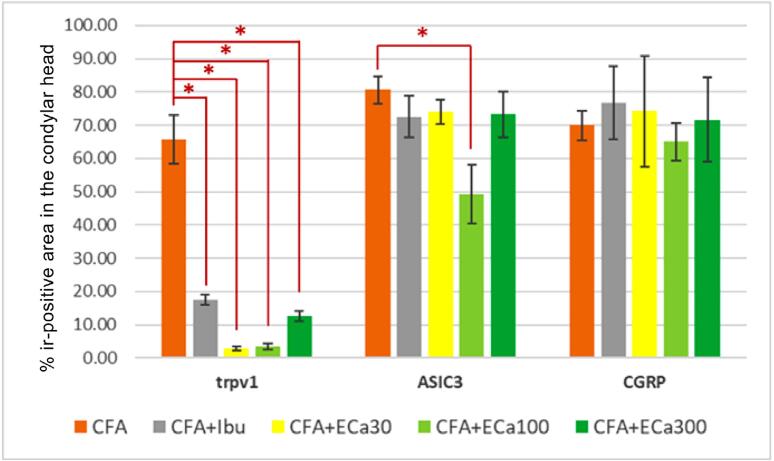
Graph showing the percentage of the ir-positive area in the condylar head of the ibuprofen and ECa 233-treated groups according to protein expression. One-way ANOVA with Dunnett’s test, n=5

The ibuprofen group and all ECa 233-treated groups showed reduced TRPV1 expression (one-way ANOVA with Dunnett’s test, n=5; F (5,24) = 297.24, *p*≤0.001). The 100 mg/kg ECa 233 group showed the protective effect of the extract against ASIC3 (one-way ANOVA with Dunnett’s test, n=5; F (5,24) = 17.56, *p*≤0.001). Meanwhile, the ibuprofen group and all ECa 233-treated groups showed no effect on CGRP expression (one-way ANOVA with Dunnett’s test, n=5; F (5,24) = 25.39, *p*≤0.001) (Figure 3).

## Discussion

The inflammatory mediators TRPV1, ASIC3, and CGRP showed increased expression in mice with TMJ-OA on day 21. ECa 233 at doses of 30, 100 and 300 mg/kg prevented this pathogenesis by modulating the local expression of TRPV1, ASIC3, and CGRP. In this study, ECa 233 treated TMJ-OA more effectively than ibuprofen.

The upregulation of inflammatory mediators, such as bradykinin, TNF-α, nerve growth factor, and IL-1β was increase in the joint inflammation. Furthermore, joint inflammation increases temperature, acidity in the inflamed area, and the development of osteoarthritis.^19-21^ Subsequently, sensitization, and upregulation of TRPV1, ASIC3, and CGRP. Our experiments confirmed that the TRPV1, ASIC3, and CGRP levels significantly increased on day 21 after injecting CFA in the TMJ.

Moreover, our study showed that both ibuprofen and different concentrations of ECa 233 (30, 100, and 300 mg/kg) failed to affect the anatomical changes in the TMJ. However, ibuprofen only significantly inhibited TRPV1. Ibuprofen inhibits inflammation via nonselective COX inhibitors. Therefore, as the inflammation decreases, TRPV1 also decreases.^22^ Ibuprofen can also inhibit TRPV1 via serotonin conjugation.^23^ Although its effect on ASIC3 is marginal, it suppresses ASIC1a— another ASIC subunit—because the ibuprofen-binding site in ASIC3 has different amino acids than ASIC1a.^9^ Moreover, TRPV1 and ASIC3 are the expressed receptors detected on the chondrocyte, whereas CGRP is a neuropeptide secreted and bound to the CGRP receptor at the subchondral area. In mice, ibuprofen can significantly decrease the serum levels of CGRP from endotoxicosis.^10,20^ However, studies evaluating CGRP expression directly on the subchondral area are still unavailable.^24^ In our study, both ibuprofen and ECa 233 failed to downregulate CGRP levels on subchondral areas. This may have happened because CGRP—which is detected on the subchondral bone—is a molecule secreted from several locations, such as nerve axons and endothelial cells, which could have traveled into the synovial fluid and bound to the receptor in the chondrocyte.

TRPV1 activation enhanced bone resorption in mice tibias and augmented C-fiber innervation in the bone.^25^ Moreover, TRPV1 knockout mice showed severe alveolar bone loss after seven days; but when TRPV1 activation was prolonged, CGRP expression decreased in the alveolar bone^10^. An *in vitro* study on CGRP showed that osteoclastogenesis inhibition suppresses bone loss.^20,25^ Therefore, ECa 233 failed to significantly change CGRP levels in this area; though this result requires further investigation.

According to our results, ECa 233 significantly inhibited TRPV1. Another research suggests that ECa 233 can inhibit the production of lipopolysaccharide-stimulated pro-inflammatory mediators, including TNF-α and IL-1β without cytotoxicity. Such production is related to the sensitization and upregulation of TRPV1.^13^ Thus, ECa 233 can reduce TRPV1 levels by this pathway. Moreover, a concentration of 100 mg/kg of ECa 233 significantly downregulated ASIC3 expression. According to another research, ECa 233 can inhibit ASIC3 by suppressing bradykinin. ECa 233 can also reduce inflammation, thereby lowering both temperature and acidity.^14^ Therefore, administering ECa 233 can reduce the expression of TRPV1 and ASIC3.

Our study also showed that while a concentration of 100 mg/kg of ECa 233 significantly downregulated ASIC3 expression, the higher concentration (300 mg/kg) showed no beneficial effect. Similarly, recent research showed that ECa 233 treatment shows an inverted U-shaped dose-response relationship.^26^ This could be because a lower concentration of ECa 233 (30 mg/kg) is insufficient to effectively inhibit ASIC3 expression, whereas a higher concentration of ECa 233 (300 mg/kg) may activate other biological pathways and no longer inhibit ASIC3 expression. Another possible reason for the latter result was the increased activity of gamma-aminobutyric acid (GABA), which reduces progressive osteoarthritis.^27^ However, GABA expression is not directly related to inflammatory markers such as ASIC3.^2,28^

Furthermore, a CFA-induced TMJ-OA mouse model has been proposed.^12^ Our study showed that the condylar head of the TMJ changed after CFA induction. The evolution of TMJ-OA caused the progressive degeneration of the TMJ, represented by a graded score up to day 21 of the experiment. Therefore, our study confirms this model by grading the TMJ diagnosed with TMJ-OA via the osteoarthritis cartilage histopathology assessment system, which is consistent with previous studies.^12,19^

Physicians prescribe anti-inflammatory drugs such as ibuprofen and corticosteroids as the standard clinical treatment of TMJ-OA.^21,29^ These drugs can prohibit the local inflammatory mediators located at the cartilage of TMJ, thereby preventing progressive TMJ-OA. However, these drugs cause side effects and are contraindicated in patients with cardiovascular diseases.^30^ Interestingly, both acute and sub-chronic toxicity tests in animal and phase I human studies showed that ECa 233 is safe.^31,32^ ECa 233 neither induced or inhibited the activities of drug-metabolizing enzymes in hepatocytes and showed no sign of toxicity in the cardiovascular system.^32^ Therefore, ECa 233 may be a potential drug of choice for TMJ-OA. However, other ECa 233 mechanisms in treating TMJ-Oa need further investigation.

## Conclusion

The 100 mg/kg concentration of Eca 233 showed a protective effect against TMJ-OA by reducing the local expression of TRPV1 and ASIC3. Due to its safe profile, it can be used as a new treatment of choice for TMJ-OA, thereby substituting NSAIDs.
